# Pattern of Antibiotic Use Among Children With Acute Respiratory Infections in Saudi Arabia: Clinical Assessment

**DOI:** 10.7759/cureus.54799

**Published:** 2024-02-24

**Authors:** Nehal Mejze Jeza Alharbi, Noha Farouk Tashkandi, Asma Mohammad Banjar, Asmaa Yassir Alotaibi, Sarah Al-Harbi, Anas Mohammed Ahmed Alqarni, Younis Abdulrahman Alharbi, Haneen H Alkenani, Abdulraoof Abdulrhman Bokhari

**Affiliations:** 1 Nursing Administration, King Salman bin Abdulaziz Medical City, Medina, SAU; 2 Medical Research, King Saud Bin Abdulaziz University for Health Sciences College of Medicine, Riyadh, SAU; 3 College of Pharmacy, Umm Al-Qura University, Makkah, SAU; 4 Pharmacy, Umm Al-Qura University, Makkah, SAU; 5 College of Medicine, Umm Al-Qura University, Makkah, SAU; 6 Medicine, Umm Al-Qura University, Al-Qunfudah, SAU; 7 Risk Management and Patient Safety Department, College of Medicine, Taibah University, Medina, SAU

**Keywords:** treatment pattern, antibiotics, pediatrics, acute respiratory infections, saudi arabia

## Abstract

Objectives: This study aimed to evaluate the common clinical diagnoses and treatment management of acute respiratory infections (ARIs) in children and determine when antibiotics are recommended and prescribed.

Methods: A retrospective review of medical charts was carried out at King Salman Bin Abdulaziz Medical City (KSAMC) Hospital to assess pediatric patients diagnosed with ARIs aged 0-14 years, excluding those requiring antibiotics for conditions other than ARIs. Data, including demographic diagnoses and treatment management, were extracted using consecutive sampling, and statistical analyses were conducted using Jamovi software.

Results: A total of 285 pediatric patients were included, with a median age of 3 (IQR = 1-6) years and a male predominance of 59.2%. Bronchopneumonia was the most common respiratory disease, diagnosed in 39.1% of participants. The median durations for illness and hospital admission were four and three days, respectively. Clinical evaluations showed an average respiratory rate of 28±10.5 breaths per minute and a mean oxygen saturation of 96.4±3.46% through pulse oximetry. The use of antibiotics was commonly prescribed in ARI patients only when accompanied by certain bacterial infections (46.32%).

Conclusions: ARIs are a common viral health issue among children, emphasizing that not all ARIs in children are caused by bacteria and that antibiotics should only be used when there is a bacterial infection present. Enhanced diagnostic precision, patient awareness, and provider education are the global community's recommendations to prevent the presence of antibiotic resistance and the irrational use of antibiotics.

## Introduction

Acute respiratory infections (ARIs) pose a significant health risk, particularly in children [1]. While many ARIs are viral and do not necessitate antibiotic treatment, there are certain bacterial respiratory infections where antibiotics are indeed required. These include conditions like sinusitis, otitis media, streptococcal pharyngitis, bronchitis, pneumonia, and tonsillitis [2-4]. However, the excessive use of antibiotics leads to the emergence of antibiotic-resistant bacteria, complicating future treatments for bacterial infections [5,6].

For the management of prevalent childhood illnesses such as ARIs, the World Health Organization (WHO) has established specific guidelines [7]. WHO recommends antibiotic use for children exhibiting severe pneumonia, rapid breathing, lower chest wall drawing, or other critical signs [2]. Similarly, the American Academy of Family Physicians (AAFP) has developed guidelines for the judicious use of antibiotics in upper respiratory tract infections (URIs). According to the AAFP, antibiotics should be avoided in cases of uncomplicated URIs, which are typically viral. However, they advocate for early antibiotic intervention in certain scenarios, such as acute otitis media, streptococcal pharyngitis, or epiglottitis [8].

Antimicrobial resistance (AMR) presents a serious global public health challenge, leading to heightened morbidity, mortality, and unsuccessful treatments due to infections from multidrug-resistant bacteria [9,10]. The root cause of this resistance is often linked to the irrational use of antibiotics, emphasizing the necessity for both healthcare providers and patients to be well-informed about the risks associated with frequent antimicrobial consumption [11].

The global prevalence of antibiotic prescriptions was reported to be high, with studies suggesting that between 20% and 50% of these prescriptions might be inappropriate or unnecessary [12,13]. In Saudi Arabia, the rate of irrational use of antibiotics was found to range between 41% and 92% [14].

Factors contributing to irrational prescriptions encompass diagnostic doubts, knowledge gaps, cultural and financial pressures, time constraints, potential legal actions, and the need to fulfill patient or parent expectations [15]. In pediatric care, antibiotics remain highly prescribed, often for acute respiratory illnesses [16]. Interestingly, while most respiratory diseases are viral and do not require antibiotics, their over-prescription is evident, leading to concerns over antibiotic use, especially in outpatient pediatric settings for conditions where antibiotics might be unnecessary [17].

Such unnecessary prescribing is a major contributor to AMR, with doctors and nurses frequently held accountable. Guidelines for rational antibiotic use and decision-support tools are used in order to improve antibiotic prescribing. There have been challenges in implementing this approach into routine clinical practice, and the current practice presents a risk of both quantity and quality errors [18]. This study aimed to assess the common clinical diagnoses and antibiotic prescription rate of ARIs in children and to determine when antibiotics are recommended and prescribed.

## Materials and methods

Study design and setting

A retrospective medical chart review was conducted at King Salman Bin Abdulaziz Medical City (KSAMC) Hospital in Madinah, Saudi Arabia. The medical records were utilized to retrieve information on pediatric patients diagnosed with ARIs. The ethical approval was obtained from the KSAMC Institutional Research Board (IRB: 023-016). A written informed consent was obtained from the parents prior to study initiation.

Participants

Children presented with ARIs were included based on the following criteria: age range from 0 to 14 years, both male and female children, and clinical diagnosis of ARIs. Any pediatric patient with clinical diagnoses mandating antibiotic treatment aside from ARIs was excluded.

Data sources and variables

Medical records served as the principal data source. The following information was extracted from these records: demographics (age, gender, region, weight, and height), clinical data (symptoms, chief complaints, and methodologies employed for diagnosis), and treatment data (specifics of the treatment approach, type of antibiotic administered, and any supplementary treatments provided).

Sample size and sampling technique

The desired sample size was determined to be 385 participants, considering a confidence level of 95% and an accepted margin of error of 5%. A consecutive sampling technique was adopted to select the participants to minimize selection bias.

Statistical analysis

All statistical analyses were performed using the Jamovi software (https://www.jamovi.org/). Descriptive statistics represented categorical data as frequencies and percentages. For continuous variables, the mean and standard deviation, or median and interquartile range (IQR), were calculated, and the Mann-Whitney test was used to test the hypothesis. The chi-square test was employed to establish relationships between demographic, clinical, and antibiotic-related data, with a P-value threshold of <0.05 set for statistical significance.

## Results

Demographic and clinical characteristics

In our study population, the median age of the children was three years, with an IQR of one to six years (mean 4.07±4.0 years). There was a higher representation of males (59.2%) compared to females (40.8%). When evaluating comorbidities, a significant portion of the participants' guardians or parents (47.9%) did not report any associated conditions. However, among those who did, the most prevalent comorbidities were epilepsy (8.9%), metabolic disorder (8.5%), and asthma (6.7%). Less common comorbidities ranged from allergies and chronic sinusitis to more severe conditions like congenital malformations of the heart and septic shock, each accounting for less than 5% of the population.

Regarding the types of respiratory diseases diagnosed, bronchopneumonia was the most prevalent, affecting 123 participants (43.3%). This was followed by acute bronchiolitis, diagnosed in 55 children (19.4%), and acute upper respiratory infection in 40 children (14.1%). Less frequent diagnoses, such as asthma and pneumonitis, were noted in fewer than 5% of the patients. The median duration of illness across the population was four days (IQR: 2-5 days), while the median duration of hospital admission was three days with an IQR of two to five days, as shown in Table 1.

**Table 1:** Demographic and clinical characteristics IQR: interquartile range, DM: diabetes mellitus, HTN: hypertension, NR: not reported, GERD: gastroesophageal reflux disease

Variables		N (%)
Age	Median (IQR)	3 (1–6)
Gender	Male	168 (59.2%)
	Female	116 (40.8%)
Comorbidities	Allergies	1 (0.4%)
	Asthma	19 (6.7%)
	Atresia	10 (3.5%)
	Benign congenital hypotonia	12 (4.3%)
	Chronic sinusitis	1 (0.4%)
	Congenital malformation of the heart	11 (3.9%)
	DM + HTN	6 (2.1%)
	epilepsy	25 (8.9%)
	GERD	1 (0.4%)
	Hydrocephalus	1 (0.4%)
	Hypertension	12 (4.3%)
	Infection	5 (1.8%)
	Neonatal jaundice	3 (1.1%)
	Obesity	1 (0.4%)
	Recurrent respiratory infections	1 (0.4%)
	Septic shock	3 (1.1%)
	Metabolic disorder	24 (8.5%)
	Not reported	135 (47.9%)
Type of respiratory disease	Acute bronchiolitis	55 (19.4%)
	Bronchopneumonia	123 (43.3%)
	Acute pharyngitis	27 (9.5%)
	Acute upper respiratory infection	40 (14.1%)
	Respiratory disorder	2 (0.7%)
	Pneumonitis	6 (2.1%)
	Respiratory infection	17 (6.0%)
	Asthma	12 (4.2%)
	Lower upper respiratory infection	2 (0.7%)
Duration of illness, days	Median (IQR)	4 (2–5)
Duration of hospital admission	Median (IQR)	3 (2–5)

Clinical diagnosis methods

The mean respiratory rate was observed to be 28±10.5 breaths per minute. Pulse oximetry revealed a mean oxygen saturation of 96.4±3.46%. Nose/throat swabs were performed on 12 individuals, representing 4.21% of the cohort, while most participants did not undergo nasal/sputum discharge tests or rapid antigen tests. Notably, all the swab tests returned negative results, with no positive cases identified, as shown in Table 2.

**Table 2:** Clinical diagnosis methods

Variable		Mean ± SD/N (%)
Respiratory rate		28±10.5
Pulse oximetry		96.4±3.46
Nose/throat swab	Performed	12 (4.21%)
	Positive	0 (0.0%)
	Negative	12 (4.21%)

Medications

The rate of antibiotic use was 46.32%. Cephalosporins were used in 36.84%, glycopeptide antibiotics in 4.92%, and carbapenems in 4.56%. The most commonly used antibiotics were ceftriaxone (34.03%), piperacillin/tazobactam (4.92%), meropenem (4.56%), and amoxicillin (2.81%).

Association between the class of antibiotic, demographic, and clinical characteristics

The study did not find a significant difference between the prescription of carbapenem-type antibiotics and cephalosporin antibiotics in terms of gender (p = 0.915), age (p = 0.063), type of respiratory disease (p = 0.626), average durations for symptoms (p = 0.626), and average hospital stays (p = 0.805), as shown in Table 3.

**Table 3:** Association between the class of antibiotic, demographic, and clinical characteristics

Variables		Carbapenem-type antibiotics	Cephalosporin antibiotics	P-value
Gender	Male	8 (61.5%)	63 (60.0%)	0.915
	Female	5 (38.5%)	42 (40.0%)	
Age		6.04±4.03	4.35±2.92	0.063
Type of respiratory disease	Acute bronchiolitis	3 (23.1%)	20 (19.0%)	0.626
	Acute pharyngitis	2 (15.4%)	13 (12.4%)	
	Acute upper respiratory infection	0 (0.0%)	15 (14.3%)	
	Bronchopneumonia	5 (38.5%)	38 (36.2%)	
	Pneumonitis	0 (0.0%)	2 (1.9%)	
	Bacterial pneumonia	2 (15.4%)	5 (4.8%)	
	Asthma	0 (0.0%)	5 (4.8%)	
	Lower upper respiratory infection	0 (0.0%)	2 (1.9%)	
	Respiratory disorder	0 (0.0%)	0 (0.0%)	
	Respiratory Infection	1 (7.7%)	5 (4.8%)	
Duration of symptoms		3.67±1.30	3.89±1.53	0.626
Hospital duration		2.69±0.95	2.75±0.85	0.805

## Discussion

The global concern over antibiotic resistance, coupled with the high prevalence of antibiotic use in pediatric populations, highlights the importance of our study. Our investigation focused on the use of antibiotics among Saudi children with ARIs, aiming to shed light on the rationality of antibiotic prescribing patterns in the presence of bacterial infections. The demographic profile of our study, dominated by a young median age of three years and a male predominance, is consistent with global pediatric cohorts diagnosed with ARIs. This demographic may be vital in interpreting antibiotic usage patterns since younger children, particularly males, are often more susceptible to respiratory infections due to their developing immune systems and narrower airways [19-21].

Comorbidities, particularly those such as epilepsy, metabolic disorders, and asthma, identified in our cohort might have influenced antibiotic prescription patterns. It was reported that patients with comorbidities were associated with 47% more antibiotic prescriptions compared with the general population [22,23]. Children with these underlying conditions might be perceived as more vulnerable, potentially leading to a more aggressive treatment approach [24].

However, it is essential to recognize that while antibiotics are indispensable for bacterial infections, they are ineffective against viral ones, which are a common cause of ARIs in children [25,26]. It is noteworthy that bronchopneumonia was the most commonly diagnosed respiratory disease in our study, followed by acute bronchiolitis and acute upper respiratory infection. Given the bacterial nature of pneumonia, its high prevalence justifies the rational use of antibiotics in our cohort. However, irrational use might arise when antibiotics are prescribed for conditions primarily caused by viruses, such as bronchiolitis [27]. Relying predominantly on clinical symptoms without objective diagnostic tools, such as electron microscopy, culture, antigen detection assays, serological tests, or nucleic acid amplification, might contribute to the potential over-prescription of antibiotics [28].

Diagnostic precision is critical in guiding rational antibiotic use; therefore, the absence of these tests might result in treatment decisions based on broad clinical symptoms rather than specific microbial etiologies [29].

Children were most frequently given penicillin (51.3%), followed by macrolides (21.9%) and cephalosporin (7.6%), and 11% were prescribed a combination of two or more antibiotics concurrently. In 1988, there were no observed instances of penicillin resistance in *Streptococcus pneumoniae* samples. Yet, by 1995, this resistance had escalated to 17%. Furthermore, 30% of these samples exhibited resistance to erythromycin and 23% to co-trimoxazole. A subsequent observation revealed that resistance levels had surged to 80% for erythromycin and 72% for co-trimoxazole [30].

In Saudi Arabia, a systematic review showed that the prevalence of antibiotic misuse among the Saudi population ranges from 41% to 92%, especially among children. This very high prevalence could be attributed to several factors, including the level of education, socio-economic status, behavioral characteristics, and cultural factors [14]. In addition, the inappropriate practices were highlighted in another Saudi cohort study; 58.1% reported inappropriate antibiotic usage. The primary rationale for antibiotic intake was due to both "viral and bacterial" causes (35%), followed by just "viral" (21%), and solely "bacterial" (20%) reasons. Furthermore, a notable 22.4% of patients could not specify the reason for their antibiotic consumption [31].

Such practices contribute significantly to the increasing prevalence of AMR in Saudi Arabia. A surveillance study in Saudi Arabia revealed that 32% of *Staphylococcus aureus* were methicillin-resistant (MRSA), while 33% of *Streptococcus pneumoniae* showed resistance to penicillin G, and 26% were resistant to erythromycin [32].

To mitigate the unwarranted use of antibiotics among children, several strategic measures are critical. Elevating awareness among parents and healthcare providers about the risks and consequences of unnecessary antibiotic usage in children is crucial. Pediatric care guidelines need refining, emphasizing diagnostic accuracy before prescribing antibiotics. Comprehensive education campaigns, especially during World Antibiotic Awareness Week, should specifically target pediatric antibiotic misuse [7]. Furthermore, implementing stringent antibiotic stewardship programs in healthcare facilities catering to children can ensure judicious antibiotic use. Collaborative efforts between healthcare professionals, parents, and educators can play a transformative role in safeguarding children's health against antibiotic overuse and its subsequent risks.

This study has some limitations, including not assessing the clinical outcomes of the children post-antibiotic treatment, which can be resource-intensive and require additional healthcare visits and tests. This might not be feasible in all settings or for all populations. Future research should aim to provide more insights into the decision-making processes of healthcare providers and investigate the long-term outcomes of antibiotic use. Finally, due to the challenges in justifying diagnoses and assessing the rationality of antibiotic use solely from records, these factors should be acknowledged as limitations of our study.

## Conclusions

ARIs frequently occur as viral illnesses in children, and it is important to note that not all ARIs are caused by bacteria. Antibiotics should only be administered when a bacterial infection is identified. Overuse of antibiotics can contribute to antibiotic resistance, and to prevent irrational prescriptions, the global community recommends improving diagnostic accuracy, raising patient awareness, and educating providers. It is crucial to follow evidence-based guidelines and indications for the appropriate use.
